# Expanding treatment options for patients with HER2+ metastatic breast cancer with margetuximab plus chemotherapy: a case report series

**DOI:** 10.3389/fonc.2024.1419246

**Published:** 2024-08-16

**Authors:** Reshma Mahtani, Natasha Harpalani, Fengting Yan, Kristen Phiel, Iuliia Kovalenko

**Affiliations:** ^1^ Miami Cancer Institute – Medical Oncology, Baptist Health South Florida, Miami, FL, United States; ^2^ Miami Cancer Institute, Baptist Hospital of Miami, Miami, FL, United States; ^3^ Memorial Sloan Kettering Cancer Alliance, Miami Cancer Institute, Miami, FL, United States; ^4^ Breast Medical Oncology, Swedish Cancer Institute, Seattle, WA, United States; ^5^ First Hill - True Family Women’s Cancer Center, Swedish Health Services, Seattle, WA, United States; ^6^ Eversana, Amherst, MA, United States; ^7^ Internal Medicine, University of Pittsburgh Medical Center (UPMC) Harrisburg, Harrisburg, PA, United States

**Keywords:** margetuximab, HER2+, metastatic breast cancer, later-line treatment, case report

## Abstract

**Background:**

Human epidermal growth factor receptor 2 protein (HER2)-positive (+) metastatic breast cancer (MBC) is an aggressive disease and patients often undergo multiple lines of therapy following HER2 targeted therapies. The most recent National Comprehensive Cancer Network (NCCN) guidelines recommend margetuximab plus chemotherapy as fourth-line or later therapy for HER2+/hormone receptor (HR) + or negative (–) MBC. The aim of this case series is to provide information regarding margetuximab utilization in clinical practice as later-line therapy in women with HER2+ MBC.

**Case summaries:**

Margetuximab plus chemotherapy was used as fourth- or later-line treatment in patients who had received multiple HER2-targeted agents, including trastuzumab, pertuzumab, ado-trastuzumab emtansine, trastuzumab deruxtecan, tucatinib, and neratinib. Patients responded to margetuximab plus chemotherapy with real-world progression-free survival (PFS) of 3, 4, and 7 months.

**Conclusion:**

Clinical outcomes from three heavily pretreated patients with metastatic HER2+/HR+ MBC demonstrated that margetuximab plus chemotherapy resulted in real-world PFS comparable to that reported in the controlled pivotal clinical trial and support use of this targeted therapy option in appropriately identified patients.

## Introduction

Human epidermal growth factor receptor 2 (HER2)-positive (+) breast cancer (BC) is a subtype of breast cancer where amplification of the *HER2* gene results in HER2 receptor overexpression, which is a major driver of tumor development and progression (1). HER2+ BC accounts for approximately 14% of total BC cases in the United States; it is highly aggressive and has a high associated risk for mortality (2–4). Overall, patients with HER2+ BC have a poor prognosis. The 5-year survivals in patients with HER2+/hormone receptor (HR) + and HER2+/HR negative (–) metastatic (M) BC are 45.6% and 39.5%, respectively (2). Management of patients with HER2+ BC was revolutionized by the advent of trastuzumab, a monoclonal antibody (mAb) targeting HER2 (5). Chemotherapy in combination with trastuzumab (+/- pertuzumab) is routinely utilized in the (neo)adjuvant setting for patients with early-stage HER2+ BC. Dual HER2 blockade with trastuzumab and pertuzumab has become part of the standard of care for women with stage II and III HER2+ BC (6–8). Nevertheless, approximately 30% of patients still experience recurrence or metastasis despite receiving treatment in the early-stage setting (9). The combination of trastuzumab plus pertuzumab and chemotherapy is a preferred first-line treatment for HER2+ MBC (10–12), but ultimately most patients experience progression of disease on this therapy (13). In fact, patients with HER2+ MBC generally go on to receive multiple lines of therapy and, with rare exceptions, HER2+ MBC remains incurable, highlighting the need for additional treatment options (14, 15).

The high rate of disease progression despite HER2-targeted therapy with trastuzumab has prompted continued development of biologics and small molecules targeting HER2 (16, 17). Fragment crystallizable (Fc)-engineering strategies have been used to customize mAbs, enhancing their cytotoxic and antitumor potencies; margetuximab was developed using this technology (18). Results from the phase 3 SOPHIA trial showed that the combination of margetuximab with chemotherapy in patients with HER2+ unresectable or MBC previously treated with HER2-directed therapies was significantly superior to trastuzumab plus chemotherapy for extending progression-free survival (PFS) (19). Median overall survival (OS) was similar to trastuzumab (20). Results from this trial supported the indication for margetuximab in combination with chemotherapy for the treatment of adult patients with HER2+ MBC who have received two or more prior anti-HER2 regimens, at least one of which was for metastatic disease (21).

Additionally, pharmacogenomic targeting in HER2+ MBC may improve outcomes for patients carrying the CD16A-F allele for the Fc-gamma receptor due to the increased affinity of margetuximab for this allele over trastuzumab. In a preplanned, exploratory analysis of SOPHIA, the margetuximab-based regimen was superior to trastuzumab plus chemotherapy in prolonging overall survival (OS) in patients with the CD16A-158FF genotype (21). The MARGOT trial (NCT04425018) is currently evaluating the role of personalized treatment of stage II-III HER2+ BC in patients with the FF or FV CD16A genotype with paclitaxel plus margetuximab and pertuzumab vs paclitaxel plus trastuzumab and pertuzumab (22).

Despite the demonstrated improvement in PFS, a favorable risk-benefit profile (20, 21, 23), and the inclusion in the NCCN guidelines for use as fourth-line or later therapy for HER2+/HR+ or – MBC (12), margetuximab plus chemotherapy may remain underutilized in clinical practice (23). This may be due to the increasing number of later-line options for the treatment of HER2+ MBC (12) and uncertainty regarding best use of margetuximab in clinical practice. The aim of the three cases presented here is to describe margetuximab use as later-line therapy for HER2+ MBC in real-world clinical practice. All patient cases have been deidentified to protect patients’ and their families’ privacy.

## Case 1: 72-year-old woman, sixth-line margetuximab plus chemotherapy, 3 months PFS

### Presentation and diagnosis

**Figure 1:**
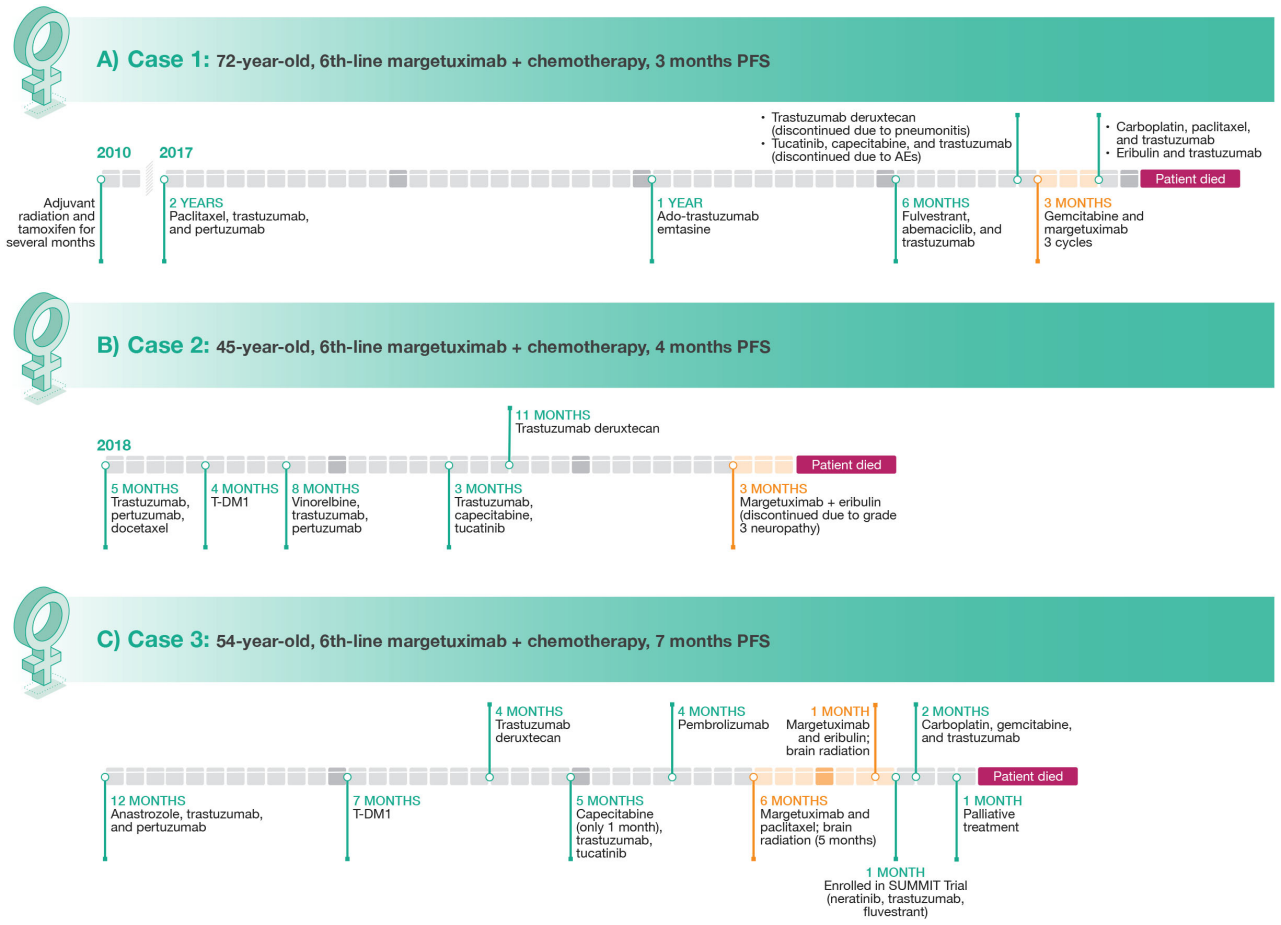
Clinical course of therapy and treatment lengths for Case 1 **(A)**, Case 2 **(B)**, and Case 3 **(C)**.

The patient was a 72-year-old woman with no evidence of a germline *BRCA* mutation, who was originally diagnosed with BC in 2010 (at age 59 years), when she underwent right lumpectomy sentinel node biopsy (**Figure 1**). Final pathology confirmed a 2 mm invasive ductal carcinoma, estrogen receptor (ER) + (95%) and progesterone receptor (PR) + (80%); HER2 status could not be determined due to insufficient tissue for testing. The decision was made to not offer adjuvant chemotherapy and trastuzumab given the overall small amount of invasive disease (pT1aN0M0). She underwent adjuvant radiation and took tamoxifen for a couple months but discontinued due to intolerance. She was followed and did well until 2017 when she reported discomfort in her low back, which led to imaging that revealed widespread bone metastases. A biopsy of the left sacrum confirmed invasive ductal carcinoma (IDC), ER+, PR–, and HER2+ (3+ by immunohistochemistry [IHC]).

### Clinical course

As first-line treatment, the patient received paclitaxel, trastuzumab, and pertuzumab which resulted in disease control for approximately 2 years. She then developed progressive bone metastases. Treatment was changed to ado-trastuzumab emtansine (T-DM1), providing disease control for approximately 12 months, at which point further progression was noted in bone and she had developed neuropathy. Treatment with fulvestrant in combination with abemaciclib and trastuzumab was given for approximately 6 months, at which point she developed symptomatic progression of disease in the bone, multiple nodal sites, and in the lung. She was then treated with trastuzumab deruxtecan. Although imaging confirmed a response to trastuzumab deruxtecan, she developed grade 2 pneumonitis that required discontinuation (24, 25). The patient then received tucatinib plus capecitabine and trastuzumab (26) but developed elevations in liver function tests that responded to dose reductions, but due to lack of appetite and intermittent diarrhea, the patient elected to discontinue treatment (27). Imaging performed in July 2022 after discontinuation of therapy demonstrated further progression of disease, with new lytic bone lesions and new metastases identified in the liver. In July 2022, the patient started treatment with gemcitabine and margetuximab. After 3 cycles, a repeat PET scan showed definitive improvement in hepatic lesions and mild improvement in bony lesions. However, this combination was stopped after the patient was hospitalized at the end of October 2022 (3 months) with suspected progression of disease and development of ascites. Treatment was then changed to carboplatin, with re-challenge of paclitaxel, in combination with trastuzumab, with no response noted on imaging. Ultimately, therapy with eribulin and trastuzumab was also not effective and unfortunately the patient succumbed to her disease.

## Case 2: 45-year-old woman, sixth-line margetuximab plus chemotherapy, 4 months PFS

### Presentation and diagnosis

The patient was a 45-year-old woman with no significant medical history who presented with a self-palpated mass in the right breast for 4 months in 2018 (**Figure 1**). A right breast biopsy indicated grade 3 ductal invasive carcinoma that was ER– and PR– with equivocal results for HER2 by IHC but positive results with fluorescent *in situ* hybridization (FISH). A staging computed tomography (CT) scan at diagnosis demonstrated a large number of liver lesions. Laboratory results indicated elevations in both alanine aminotransferase and aspartate aminotransferase. The diagnosis for this patient was *de novo* HER2+/HR– MBC (HER2 low) with liver metastases.

### Clinical course

First-line treatment was trastuzumab plus pertuzumab and docetaxel (THP) as recommended by NCCN (12) and supported by results of the CLEOPATRA trial (10). Monitoring during treatment consisted of laboratory assessment every 3 weeks, periodic cardiac function evaluation, and a CT scan. After 5 months, the patient progressed on THP and was switched to T-DM1 (28) and remained on this antibody-drug conjugate for 4 months until progression. Vinorelbine plus trastuzumab and pertuzumab was used in third line for 8 months (29), trastuzumab plus capecitabine and tucatinib in fourth line for 3 months (26), and trastuzumab deruxtecan in fifth line for 11 months (30). After disease progression, the patient was switched to margetuximab plus eribulin (19). She responded to this treatment and was stable on the regimen for 4 months. She then developed grade 3 neuropathy and treatment was discontinued.

## Case 3: 54-year-old woman, sixth-line margetuximab plus chemotherapy, 7 months PFS

### Presentation and diagnosis

A 54-year-old woman with no significant family or social history potentially related to BC was initially diagnosed with clinical stage IV (cT2cN1(f)M1) IDC of the right breast that was ER+/PR+ (90%/75%) and HER2+ (>30%) as well as high-grade ductal carcinoma *in situ* of the right breast (**Figure 1**). Whole-body PET/CT scan also revealed multiple osseous metastatic lesions. Lumbar lesion biopsy confirmed ER+/PR+ (90%/80%) HER+ (30%) MBC.

### Clinical course

The patient refused neoadjuvant chemotherapy and completed a 12-month course of anastrozole plus trastuzumab and pertuzumab (31). She then underwent palliative mastectomy of the right breast, prophylactic mastectomy of the left breast, and right axillary node excision. Pathology revealed multifocal grade 3 IDC with skin, skeletal muscle, and lymphovascular invasion. Following initial treatment, the patient underwent a 7-month course of therapy with T-DM1. Repeat PET/CT showed disease progression with hypermetabolic lesions in the neck and right axillary and mediastinal lymph nodes as well as in the right pectoralis muscle and new skeletal lesions. The patient was then switched to trastuzumab deruxtecan (32), which was discontinued 4 months later due to disease progression indicated by a PET/CT scan that revealed F-fluorodeoxyglucose (FDG)-avid supraclavicular and mediastinal lymph nodes as well as multiple soft tissue nodules along the right chest wall. The patient was then started on a combination therapy with capecitabine, trastuzumab, and tucatinib (27). Capecitabine was withheld one month later during palliative chest wall radiation therapy. A CT bone scan 5 months later showed progressive adenopathy including new lesions in the right paratracheal and right hilar lymph nodes. Foundation one genetic testing was performed and revealed microsatellite (MS)-stability, tumor mutational burden of 10 mutations per megabase, amplification in *AKT*3*, IKBKE*, *MDM4*, *PIK3C2B*, and *RAD21*; mutations in *CDC73* (W43) and *PIK3CA* (E453del, C420R); and *EED* (NM_003797) rearrangement in exon 9. Molecular profiling testing revealed that tumor tissue was ER+ (90%), amplified for ERBB2 (HER2/neu), PR+, programmed death ligand 1-positive (IHC; Sp142), MS, *NTRK* fusion negative, *AR* mutation positive, *BRCA1* and *BRCA2* negative, *PIK3CA* mutated, and *PTEN* mutation positive. The patient was started on pembrolizumab and refused chemotherapy at that time. She developed disease progression after 4 months.

At this point, it was decided to start the patient on margetuximab in combination with paclitaxel as her sixth line of therapy. Her only adverse event on this regimen was a grade 1 elevation in liver enzymes. She underwent brain magnetic resonance imaging (MRI) one month later, which revealed 5 to 6 subtentorial enhancing masses. She then underwent brain radiation therapy along with right femoral neck therapy. One month later, the patient had a positive treatment response with left cervical lymphadenopathy size reduction. Her only side effect was fatigue. Follow-up PET/CT scan revealed resolution of multiple FDG-avid uptake areas, including the parotid gland, neck, supraclavicular fossa, axilla, hilar areas, and mediastinum as well as the right adrenal gland and retroperitoneal nodules. Unfortunately, brain MRI one month later revealed multiple new enhancing lesions. Her PET/CT scan after an additional 4 months showed disease progression with new hypermetabolic lymph nodes in the neck, axilla, mediastinum, and right hilum along with new hypermetabolic foci in the skeleton and chest skin. The patient was offered alternative treatments but preferred to remain on margetuximab. Paclitaxel was discontinued and the patient was started on a combination of margetuximab with eribulin. She also underwent repeat brain radiation therapy. Unfortunately, with the addition of eribulin, the patient developed nausea, vomiting and fatigue prompting emergency department (ED) visits. CT of the abdomen performed in the ED showed worsening metastatic disease. The patient also reported recurrence of right chest wall nodules. Eribulin was discontinued one month later and margetuximab was discontinued after a total of 7 months due to disease progression. The patient then was enrolled in the SUMMIT trial and was treated with neratinib plus trastuzumab and fulvestrant (33) for one month, which was changed to a combination of carboplatin, gemcitabine, and trastuzumab one month later due to disease progression. Brain MRI 2 months later showed disease progression. Due to poor overall prognosis, the patient elected to proceed with palliative treatment. The patient died one month later.

## Discussion

Results from the patients included in these case reports indicate that patients with HER2+ MBC are likely to receive many lines of treatment. This is consistent with large scale reviews. Assessment of 59 patients with HER2+ MBC treated at a single academic center indicated that 40% of patients received at least 5 lines of treatment that included chemotherapy and >10% received at least 10 lines (34). A more recent larger study of 1390 patients with HER2+ MBC indicated 39.6% of patients received at least 4 lines of treatment (35).

The results for these cases also underscore the difficulties involved in sequencing later lines of treatment for HER2+ MBC. It is recognized that optimal sequencing of anti-HER2 agents in patients with advanced HER2+ BC is essential for maximizing the benefit of each line of treatment and slowing the progression of metastases (36). However, there are several NCCN-recommended therapies for HER2+ advanced/MBC (12), each possessing different mechanisms of action and safety profiles. Deciding on the best treatment sequencing for an individual patient is a significant challenge (36). Importantly, evidence-based recommendations to guide sequencing in later lines of therapy are lacking (12). This is reflected by the treatment sequencing for the three patients described in this paper. First-line treatment for all three patients included chemotherapy in combination with trastuzumab and pertuzumab, consistent with NCCN recommendations based on the results from the landmark CLEOPATRA trial (10, 11). Second-line treatment for each patient included T-DM1 which was the standard at the time these patients were treated (based on the EMILIA trial), but has recently been replaced by trastuzumab deruxtecan based on results from DESTINY-Breast03 (12, 28, 37). Treatment after progression on T-DM1 varied.

The combination of margetuximab and chemotherapy was used as sixth-line therapy in these cases; it provided results consistent with those from the phase 3 SOPHIA trial supporting its approval by the US Food and Drug Administration. This study included 536 patients with HER2+ BC (metastatic in ~98% of patients). Similar to the patients described in these case studies, all patients enrolled in SOPHIA had received trastuzumab, all but one had received prior pertuzumab, and 91.2% had received prior T-DM1. One-third of the patients in the trial had received ≥2 prior lines of treatment (19). The median PFS for margetuximab plus chemotherapy in SOPHIA was 5.8 months (19); real-world PFS for the 3 patients described in this report was 3, 4, and 7 months.

Similar results were seen with earlier use. Results from a recently published case study of a patient initially diagnosed with HER2+/HR– IDC that metastasized to the liver after one cycle of chemotherapy who received margetuximab plus capecitabine as fourth-line treatment indicated PFS of 7 months with this regimen (38). Another case study reported a patient with HER2+ histological grade III MBC and IDC who developed bone and liver metastases who experienced a complete response that was sustained for at least 6 months (the last evaluation reported) after receiving third-line treatment with margetuximab (39).

In the presented cases, two of the patients treated with margetuximab plus eribulin had clinically important adverse events, neuropathy in one and nausea, vomiting, and fatigue with severity that prompted ED visits in another. The extent to which margetuximab or eribulin contributed to these events is not clear. Results from a phase 1 study in which margetuximab was delivered as monotherapy to patients with advanced HER2+ solid tumors indicated no occurrences of neuropathy. Fatigue, nausea, and vomiting were reported in 24%, 29%, and 24% of patients, respectively, but none of these events were grade ≥3 in severity (40). Review of safety data for eribulin in patients with BC indicated that peripheral neuropathy occurred in 28.5% of patients (grade 3/4 in 1.5%) (41). Fatigue was reported for 23.7% of patients and nausea in 35.7% (41).

Gaining information regarding the clinical benefits and risks of margetuximab in the real world is important for several reasons. First, there is no clear choice for systemic therapy after progression on third-line treatment in patients with recurrent unresectable or metastatic HER2+ MBC. Additionally, it has been reported that results achieved with cancer therapy in the real world often fall short of those reported in controlled clinical trials (42) [compare results from Verma et al. (28) with those from Nakayama et al. (43)]. The results demonstrated in this small series of patients treated with margetuximab and chemotherapy are therefore reassuring. The case studies described here and in other recent publications (38, 39) suggest that the efficacy and safety of margetuximab in routine clinical practice are comparable to those reported in controlled clinical trials (19). This supports the view that margetuximab plus chemotherapy is a viable choice for fourth- or later-line therapy even though new additional single agents and combinations now may be used prior. This conclusion is consistent with the place in therapy for margetuximab as recommended in the NCCN treatment guidelines (12).

The results presented and reviewed underscore the importance of real-world evidence as a complement to data from controlled clinical trials. The importance of real-world experience is well established, as these observations often involve patients with disease characteristics, comorbidities, and complications that would result in their exclusion from clinical trials (44). While results from real-world clinical experience may not match that from controlled clinical trials (45), understanding the efficacy and safety of a therapy across the range of patients in which it might be used is one of multiple factors that should be considered in optimizing treatment selection. Others include patient goals and preferences, their prioritization of efficacy vs risk for adverse events, favored route of administration, and impact on quality of life (46).

While real-world results including those from case studies can complement those from randomized controlled trials, uncontrolled observations do have important limitations including that they are inherently prone to bias in selection of therapy for specific patients and confounded by factors typically controlled for in clinical trials (47). Further, the ability to generalize results from very small patients samples to larger populations is limited (48).

Going forward, selection of margetuximab for treatment may be based on genomic testing, as ongoing trials seek to clarify the role for upfront allelic variation testing. Results from the SOPHIA trial suggests the importance of determining the CD16A genotype in candidates for margetuximab treatment (21), and the use of genotyping is being evaluated prospectively in the ongoing MARGOT study (22).

## Data Availability

The original contributions presented in the study are included in the article. Further inquiries can be directed to the corresponding author.
